# Supplementary material for: Complex host/symbiont integration of a multi-partner symbiotic system in the eusocial aphid Ceratovacuna japonica
Source: iScience. 2022 Nov 2;25(12):105478. doi: 10.1016/j.isci.2022.105478 (PMC9672956; doi:10.1016/j.isci.2022.105478)
Supplement: Document S1. Figures S1–S7 [file mmc1.pdf]

iScience, Volume *25*

Supplemental information

Complex host/symbiont integration of a multi-partner symbiotic system in the eusocial aphid *Ceratovacuna japonica*

Shunta Yorimoto, Mitsuru Hattori, Maki Kondo, and Shuji Shigenobu

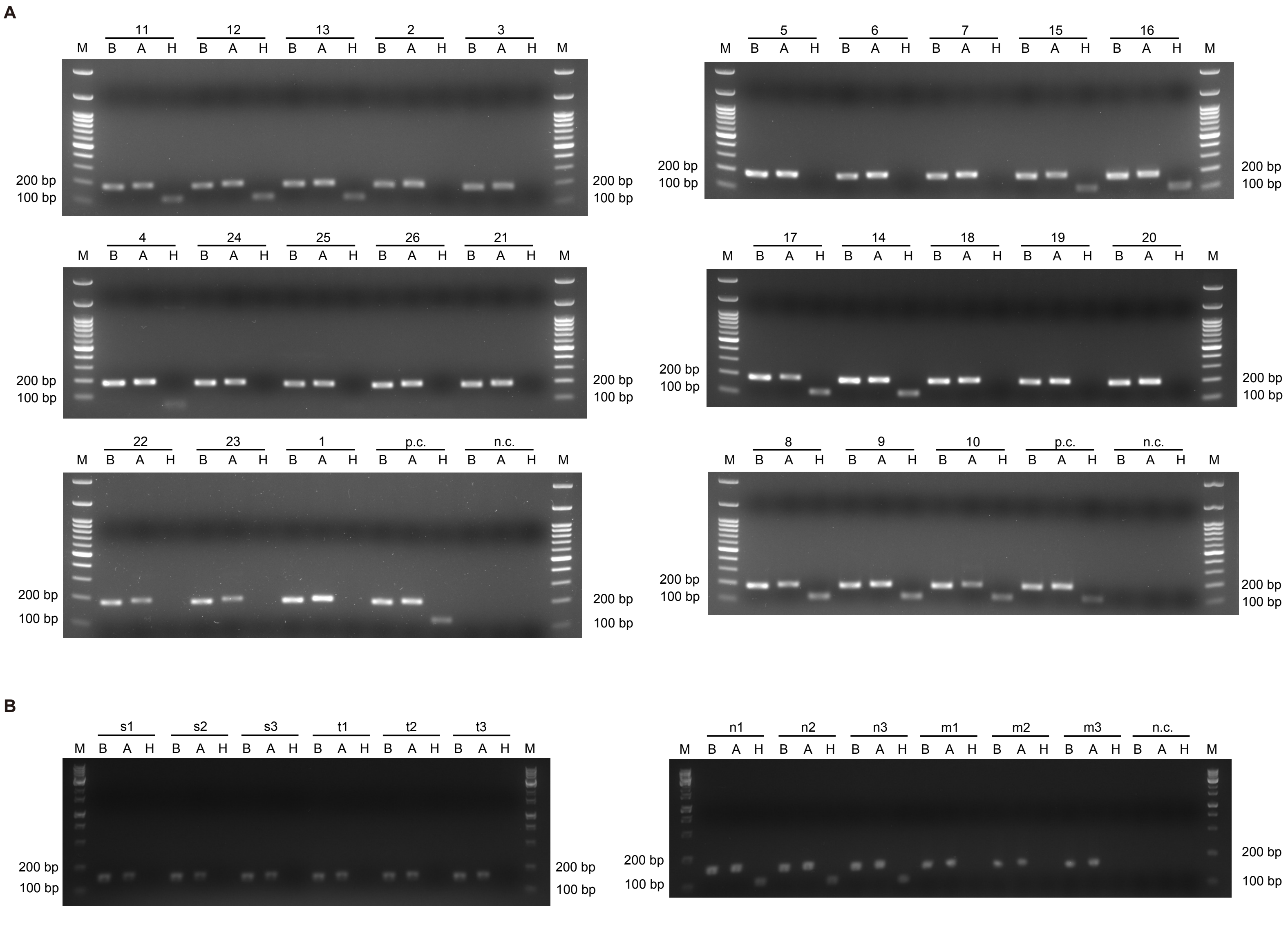

Figure S1. Diagnostic PCR detection of *Buchnera*, *Arsenophonus*, and *Hamiltonella* of *Ce. japonica*, related to Figure 1.

(A) Symbiont detection in *Ce. japonica* populations. Each sample ID number corresponds to that of Figure 1B–D.

(B) Symbiont detection in *Ce. japonica* individuals. Samples of s1–3, t1–3, n1–3, and m1–3 were derived from Sakata-koen (populations #2–4 in Figure 1), Tsushima (#24–26), Norikura (#14), and Matsudaira (#18–20), respectively.

B, A, H, M, p.c., and n.c. means *Buchnera*, *Arsenophonus*, *Hamiltonella*, DNA size marker, positive control, and negative control, respectively. Each bacterial symbiont was detected using specific primers targeting a single-copy gene, *dnaK*. PCR product sizes of *dnaK* of *Buchnera*, *Arsenophonus*, and *Hamiltonella* are 186, 192, and 117 bp, respectively. DNA size markers shows 3,000, 1,500, 1,000, 900, 800, 700, 600, 500, 400, 300, 200, and 100 bp, from top to bottom.

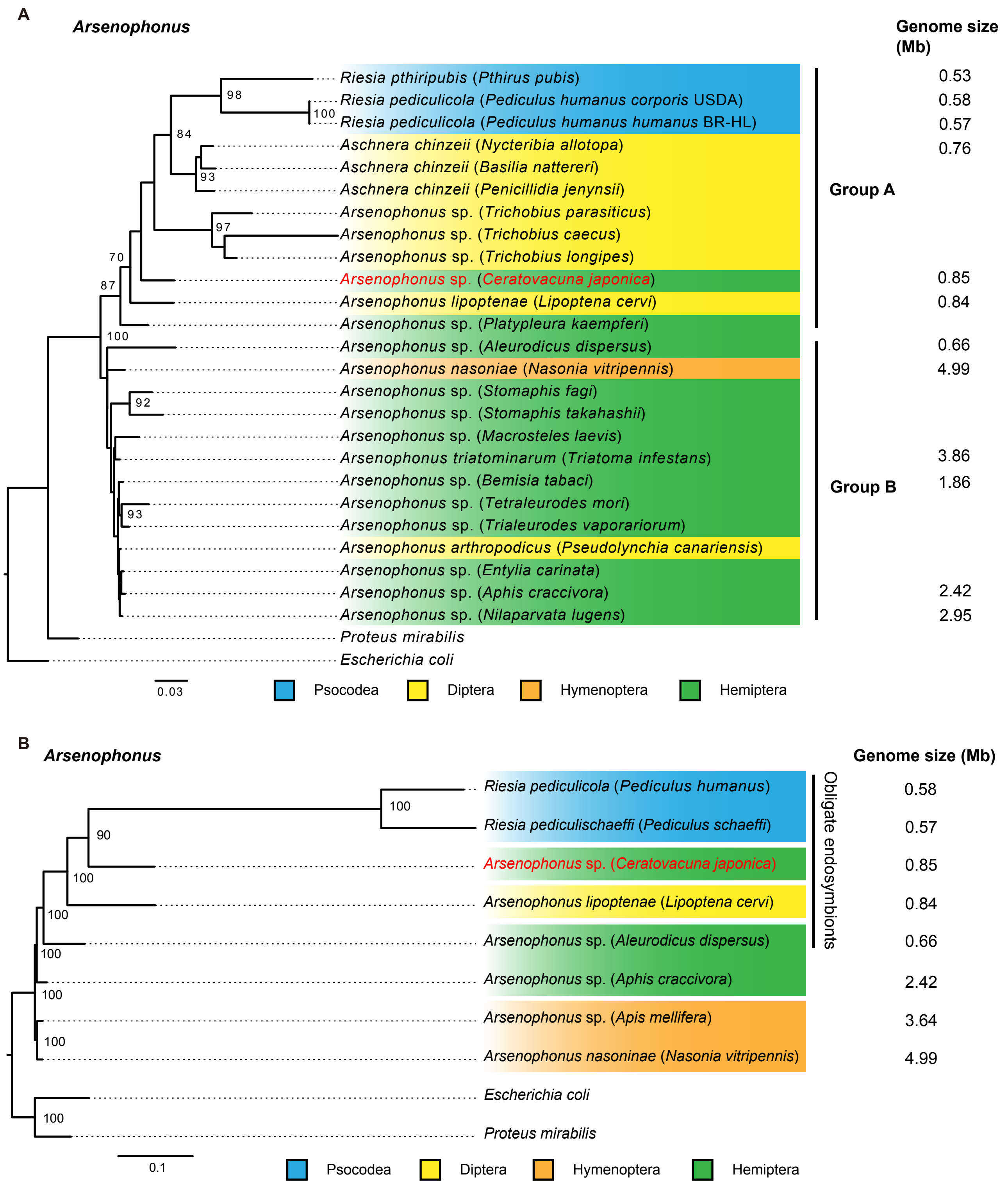

Figure S2. Phylogenetic analysis of *Arsenophonus*, related to Figure 2.

(A) Maximum likelihood (ML) tree of *Arsenophonus* based on the length of 1,451 bp of 16S rRNA sequences. *Escherichia coli* and *Proteus mirabilis* were used as outgroups.

(B) ML tree of *Arsenophonus* based on concatenated 204 single-copy orthologous protein sequences composed of 54,777 amino acid residues with recoding by the Dayhoff6 matrix. *E. coli* and *P. mirabilis* were used as outgroups. Target symbionts in this study are highlighted in red on each tree. Bootstrap values no less than 70% are indicated on each node. Scale bars represent 0.03 and 0.1 substitutions per site.

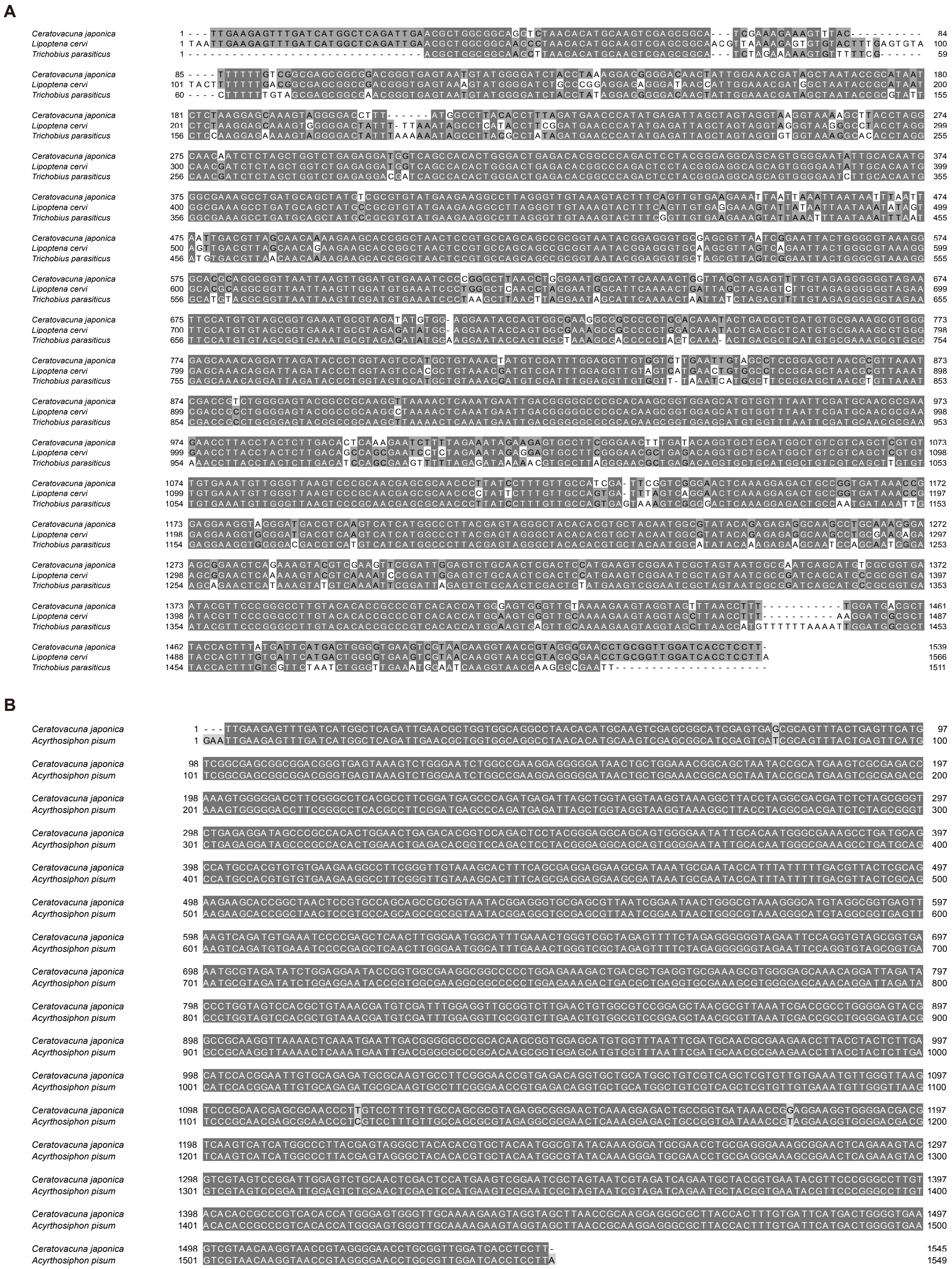

Figure S3. Alignments of 16S rRNA sequences of *Arsenophonus* and *Hamiltonella*, related to Figure 2.

(A) A multiple alignment of *Arsenophonus* symbionts of *Ce. japonica*, *L. cervi*, and *T. parasiticus* is shown. Sequence identities of *Arsenophonus* symbionts between *Ce. japonica* and *L. cervi*, and those between *Ce. japonica* and *T. parasiticus* are 93.0% and 89.4%, respectively.

(B) A pair-wise alignment of *Hamiltonella* symbionts of *Ce. japonica* and *A. pisum*. The sequence identity between these two sequences is 99.8%.

See Table S3 for the accession number of 16S rRNA sequences used for these alignments.

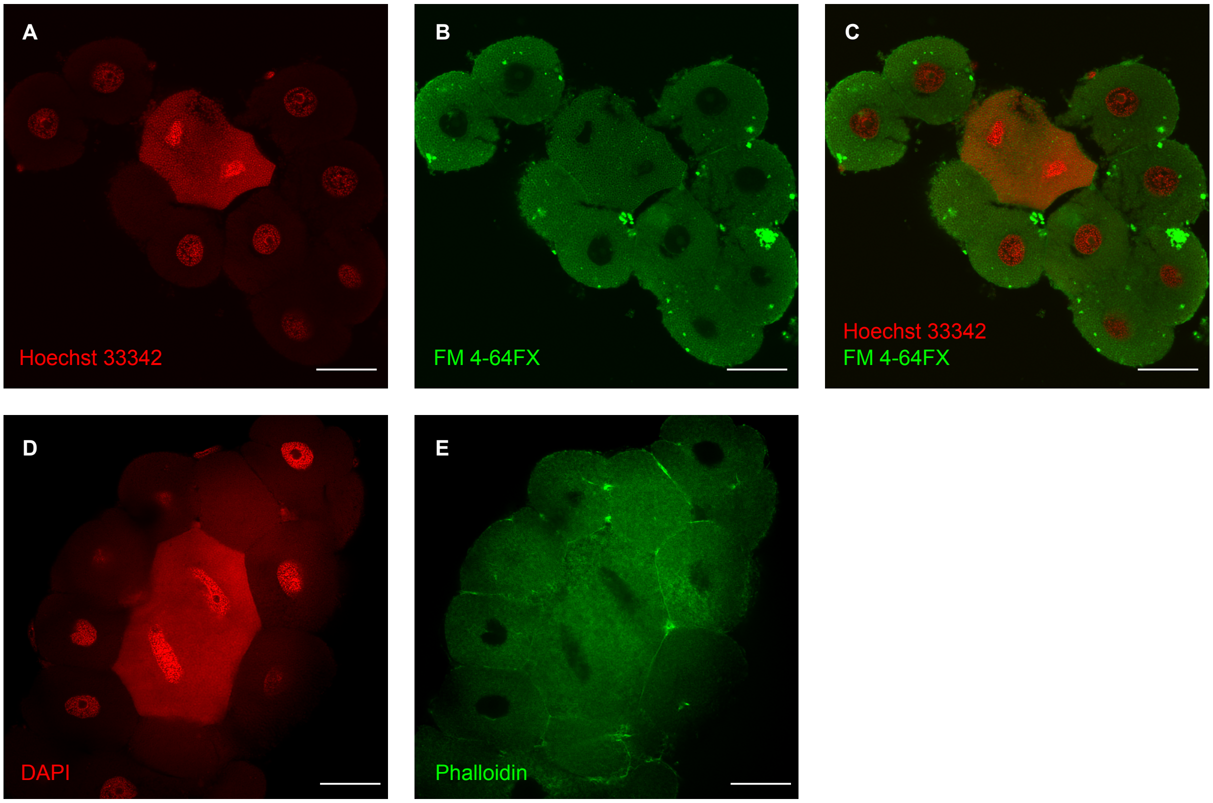

Figure S4. Structure of bacteriome, related to Figure 3.

Confocal microscopy images of bacteriomes are shown.

(A) Hoechst 33342 signals.

(B) FM 4-64FX signals.

(C) A merged image of (A) and (B).

(D) DAPI signals.

(E) Phalloidin signals.

Scale bars show 50 μm in (A–E).

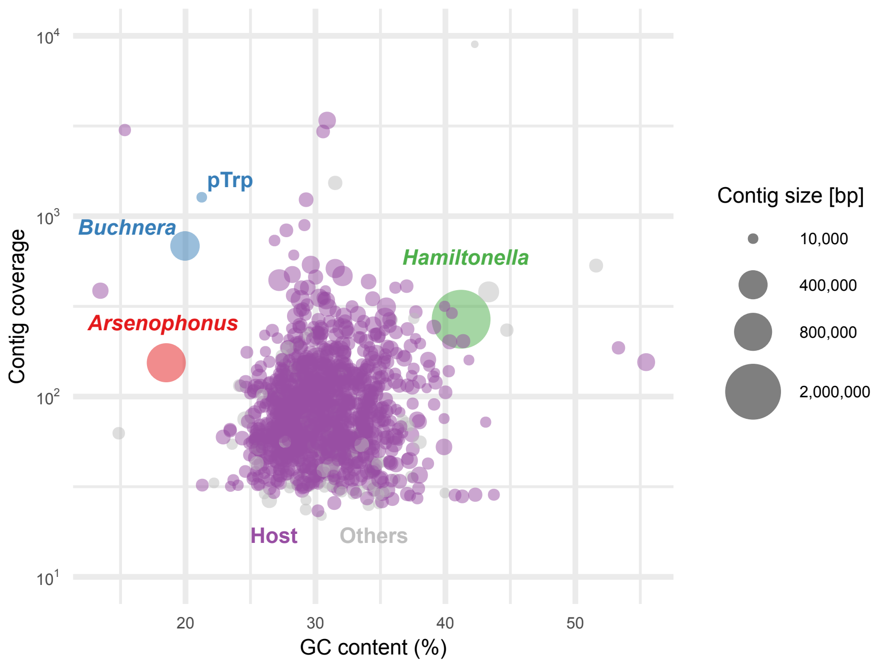

Figure S5. Taxon-annotated GC-coverage plot for metagenomic assembly, related to Figure 4.
Each blob indicates the contigs in the assembly. The blob colors are based on the best match to the taxonomically annotated RefSeq protein database using DIAMOND. Annotated contigs as “Arthropoda” were shown as the label “Host” and the label “Others” means contigs do not hit *Buchnera*, *Arsenophonus*, *Hamiltonella*, or Arthropoda. The size of the blobs indicates the length of the contigs. *Buchnera* pLeu plasmid was not found in the initial assembly used in this analysis.

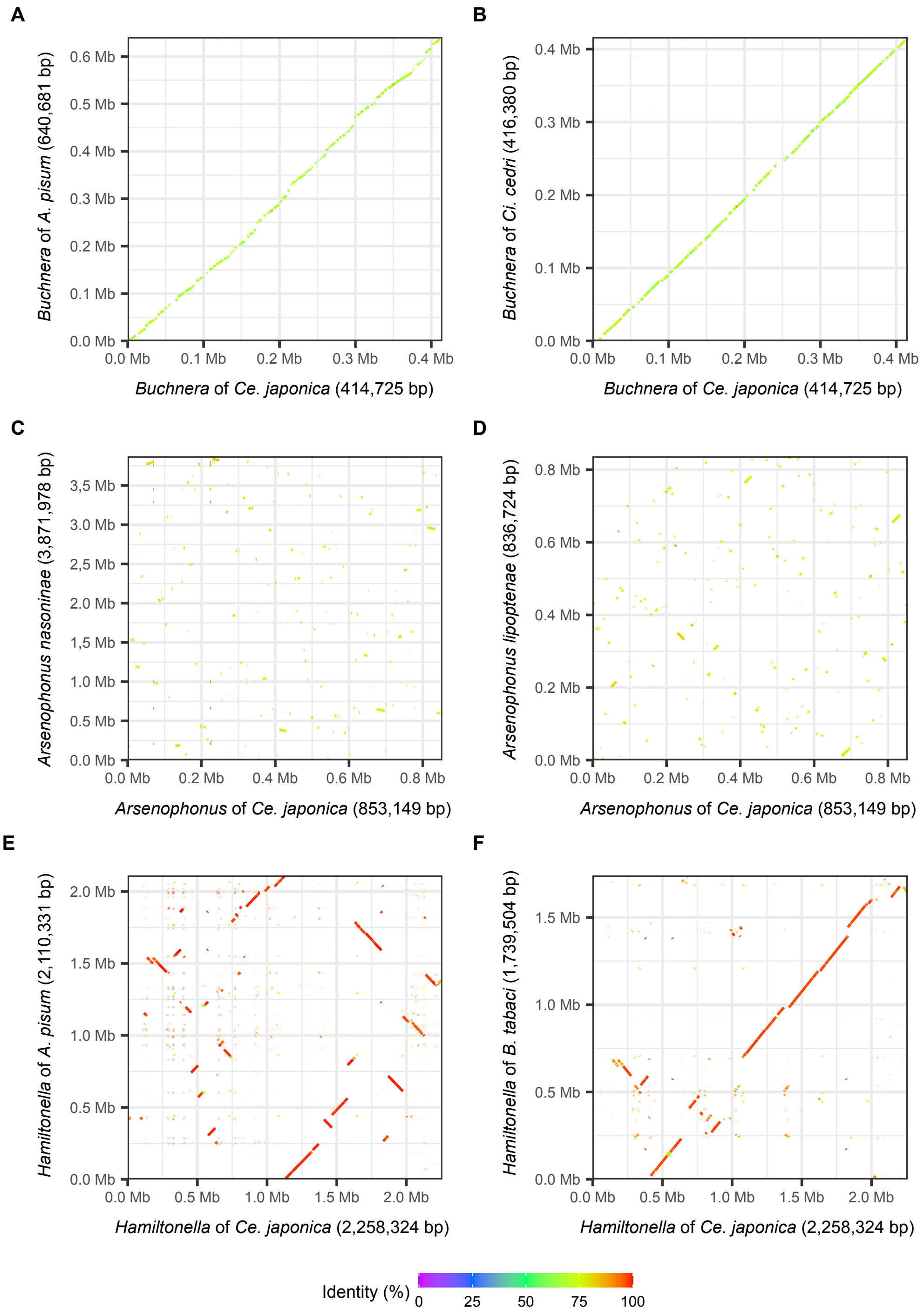

Figure S6. Synteny plots of chromosomes of *Buchnera*, *Arsenophonus*, and *Hamiltonella*, related to Figure 4.

Genomic synteny between symbionts is visualized.

(A) *Buchnera* of *Ce. japonica* and *A. pisum* (BA000003.2). The synteny is well conserved among the two *Buchnera* genomes, whereas several gaps are discernible.

(B) *Buchnera* of *Ce. japonica* and *Ci. cedri* (CP000263.1). The synteny is well conserved, whereas several gaps are discernible.

(C) *Arsenophonus* of *Ce. japonica* and *N. vitripennis* (CP038613.1). Little syntenic blocks are recognizable.

(D) *Arsenophonus* of *Ce. japonica* and *L. cervi* (CP013920.1). Little syntenic blocks are recognizable.

(E) *Hamiltonella* of *Ce. japonica* and *A. pisum* (CP001277.1). Syntenic blocks are observed with a number of inversions and rearrangements.

(F) *Hamiltonella* of *Ce. japonica* and *B. tabaci* (CP016303.1). The synteny looks well conserved with some deletions and inversions.

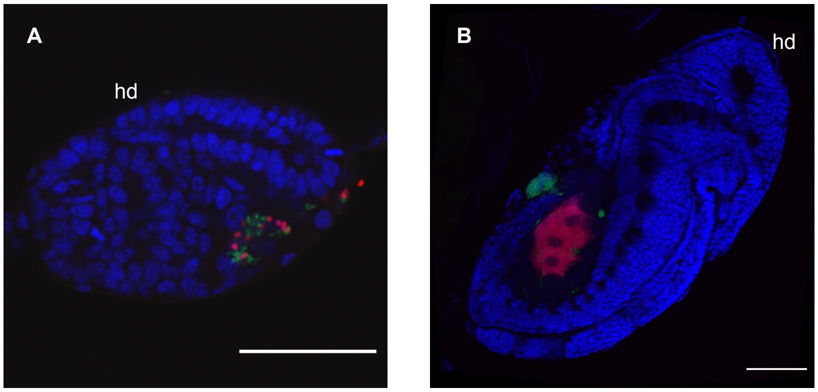

Figure S7. The infection process of *Hamiltonella* during embryogenesis, related to Figure 5.

(A) The developmental stage of an S-shape embryo. *Hamiltonella* is also infected into the embryo from the posterior part with *Arsenophonus*.

(B) The developmental stage of early germ band retraction after katatrepsis. Masses of *Hamiltonella* are observed around the bacteriome.

(A and B) Blue (DAPI), green (Cy5), and red (Cy3) indicate nuclei, *Hamiltonella*, and *Arsenophonus*, respectively. Scale bars show 50 μm in (A and B). hd, head.
